# 5-(4-Fluoro­phen­yl)-1-[4-(4-methyl­phen­yl)thia­zol-2-yl]-3-[4-(prop-2-yn­yloxy)phen­yl]-4,5-di­hydro-1*H*-pyrazole

**DOI:** 10.1107/S2414314622010033

**Published:** 2022-10-25

**Authors:** Sreeramapura D. Archana, Holalagudu A. Nagma Banu, Balakrishna Kalluraya, Hemmige S. Yathirajan, Rishik Balerao, Ray J. Butcher

**Affiliations:** a Department of Studies in Chemistry, University of Mysore, Manasagangotri, Mysore 570 006, India; b Department of Studies in Chemistry, Mangalore University, Mangalagangotri, Mangalore 574 199, India; c Thomas Jefferson High School for Science and Technology, 6560 Braddock Rd, Alexandria, VA 22312, USA; d Department of Chemistry, Howard University, 525 College Street NW, Washington, DC 20059, USA; Goethe-Universität Frankfurt, Germany

**Keywords:** crystal structure, fluoro­phen­yl, 4-(prop-2-yn­yloxy)phen­yl, pyrazole, thia­zole

## Abstract

In the title compound, C_28_H_22_FN_3_OS, four rings are almost coplanar, with the fluorophenyl ring substantially twisted. In the extended structure, aromatic π–π stacking inter­actions between the pyrazole ring and the tolyl ring link the mol­ecules into centrosymmetric dimers.

## Abstract 2

The structure of 5-(4-fluoro­phen­yl)-1-[5-(4-methyl­phen­yl)thia­zol-2-yl]-3-[4-(prop-2-yn­yloxy)phen­yl]-4,5-di­hydro-1*H*-pyrazole was determined by X-ray crystallography.

## Graphical abstract

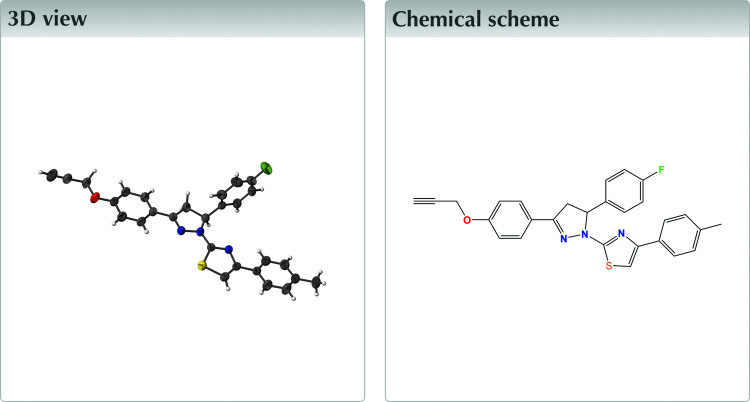

## Structure description

Pyrazoles and thia­zoles are important scaffolds in developing target drug mol­ecules. They are five-membered nitro­gen heterocycles possessing a variety of pharmacological activities, including anti­bacterial (Tanitame *et al.*, 2004 ▸), anti­fungal (Hassan, 2013 ▸), anti-inflammatory (Farghaly *et al.*, 2000 ▸), anti­depressant (Secci *et al.*, 2011 ▸), anti-analgesic (Jamwal *et al.*, 2013 ▸), anti­cancer (Keter & Darkwa, 2012 ▸), anti­tubercular (Kumar *et al.*, 2020 ▸), anti­viral (Rashad *et al.*, 2008 ▸) and anti­diabetic (Datar & Jadhav, 2014 ▸). The design, efficient synthesis and mol­ecular docking of some novel thia­zol­yl–pyrazole derivatives as anti­cancer reagents have been reported (Sayed *et al.*, 2019 ▸). We have recently reported the formation of 1-(thia­zol-2-yl)-4,5-di­hydro­pyrazoles from simple precursors, as the synthesis, spectroscopic characterization and the structures of an inter­mediate and two products (Mahesha *et al.*, 2021 ▸).

A new series of 1,3-thia­zole integrated pyrazoline scaffolds have been synthesized and characterized [Cambridge Structural Database (CSD; Groom *et al.*, 2016 ▸) refcodes DADQIL and DADQEH; Salian *et al.*, 2017 ▸]. The synthesis, fluorescence, TGA and crystal structure of a thia­zol­yl–pyrazoline derived from chalcones has been described (JUNRAN; Suwunwong *et al.*, 2015 ▸). In addition, the follow­ing crystal structures of related compounds have been reported: 2-[3-(4-bromo­phen­yl)-5-(4-fluoro­phen­yl)-4,5-di­hydro-1*H*-pyrazol-1-yl]-4-phenyl-1,3-thia­zole (IDOMOF; Abdel-Wahab *et al.*, 2013*c* ▸), 2-[5-(4-fluoro­phen­yl)-3-(4-methyl­phen­yl)-4,5-di­hydro-1*H*-pyrazol-1-yl]-4-phenyl-1,3-thia­zole (MEWQUC; Abdel-Wahab *et al.*, 2013*a* ▸), 2-[3-(4-chloro­phen­yl)-5-(4-fluoro­phen­yl)-4,5-di­hydro-1*H*-py­razol-1-yl]-4-phenyl-1,3-thia­zole (WIGQIO; Abdel-Wahab *et al.*, 2013*b* ▸), 2-[3-(4-chloro­phen­yl)-5-(4-fluoro­phen­yl)-4,5-di­hy­dro-1*H*-pyrazol-1-yl]-8*H*-indeno­[1,2-*d*]thia­zole (WOC­FEC; El-Hiti *et al.*, 2019 ▸) and 2-[3-(4-bromo­phen­yl)-5-(4-fluoro­phen­yl)-4,5-di­hydro-1*H*-pyrazol-1-yl]-8*H*-indeno­[1,2-*d*]thia­zole (PUVVAG; Alotaibi *et al.*, 2020 ▸).

Keeping this in mind, the present study was planned to synthesize a ring system that contains both pyrazole and thia­zole in a single hybrid mol­ecule with an acetyl­ene substit­uent, which can further be modified into highly functionalized heterocycles (Larock & Yum, 1991 ▸; Sonogashira, 2002 ▸).

**Figure 1:**
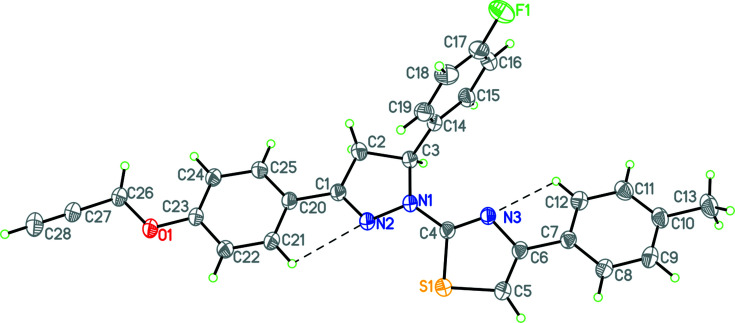
Perspective view showing the mol­ecule and atom labelling. Intra­molecular C—H⋯N inter­actions are shown as dashed lines. Displacement ellipsoids are drawn at the 30% probability level.

We now describe the synthesis and structure of the title compound, 5-(4-fluoro­phen­yl)-1-[5-(4-methyl­phen­yl)thia­zol-2-yl]-3-[4-(prop-2-yn­yloxy)phen­yl]-4,5-di­hydro-1*H*-pyrazole; the mol­ecule crystallizes in the space group *P*2_1_/*c* with one mol­ecule in the asymmetric unit (Fig. 1 ▸). The four rings that make up the central core (*A*: phenyl ring C7–C12, *B*: the five-membered ring containing atom S1, *C*, the five-membered ring containing atoms N1 and N2, *D*: phenyl ring C20–C25) are almost co-planar, the dihedral angle between *A* and *D*, which shows the overall twist, is 3.65 (7)°, that between *A* and *B* is 12.27 (7)°, that between *B* and *C* is 3.26 (5)°, and that between *C* and *D* is 0.34 (7)°. Ring *C*, which contains the *sp* ^3^ atoms C2 and C3,is almost planar (r.m.s. deviation = 0.006 Å), which we find surprising given the potential steric interactions of the H atoms connected to C2 and C3. The fluoro­phenyl substituent makes a dihedral angle of 87.84 (5)° with ring *C*.

**Figure 2:**
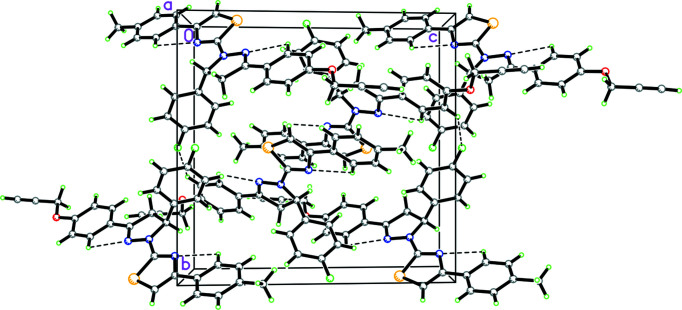
Packing diagram showing inter­molecular C—H⋯S and C—H⋯F inter­actions, as well as intra­molecular C—H⋯N inter­actions, as dashed lines.

In the crystal, there are π–π inter­actions between rings *A* and *C*, which link the mol­ecules into a centrosymmetric dimer (centroid–centroid distance = 3.649 Å, with a slippage of 0.765 Å; Fig. 2 ▸). In addition there are weak C—H⋯F and C—H⋯S interactions, which link the molecules into a three-dimensional array (see Fig. 2 ▸ and Table 1 ▸).

## Synthesis and crystallization

1-(*p*-Propyl­oxyphen­yl)-3-(4-flurophen­yl)prop-2-en-1-one (**A**) was obtained by the base-catalysed condensation of *p*-pro­pyl­yoxyaceto­phenone (3 g, 0.0174 mol) with 4-flurobenz­aldehyde (2.59 g, 0.020 mol) in an etha­nol medium employing sodium hydroxide as catalyst. Pro­panone (**A**) (2 g, 0.0075 mol), on treatment with thio­semicarbazide (1.3 g, 0.015 mol) in alcoholic potassium hydroxide, gave 3-(4-fluo­ro­phen­yl)-5-[4-(prop-2-yn­yloxy)phen­yl]-4,5-di­hydro-pyrazole-1-carbo­thio­amide (**B**).

**Figure 3:**
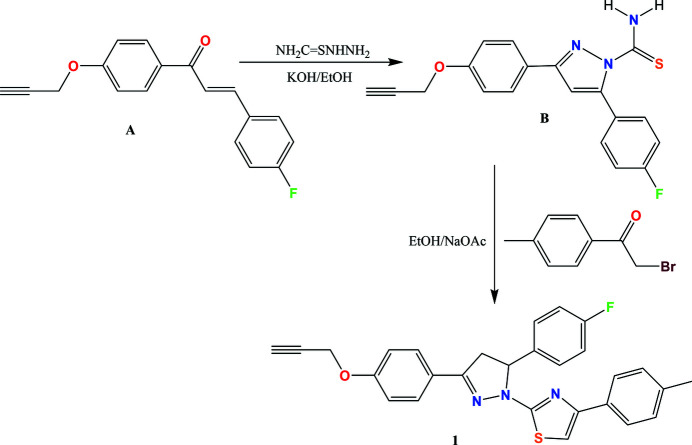
Reaction scheme for the synthesis of 5-(4-fluoro­phen­yl)-1-[5-(4-methyl­phen­yl)thia­zol-2-yl]-3-[4-(prop-2-yn­yloxy)phen­yl]-4,5-di­hydro-1*H*-pyrazole.

The synthesized **B** (1 g, 0.002 mol) and 4-methyl­phenacyl bromide (0.58 g, 0.002 mol) were added to ethanol (20 ml) and heated at reflux for 1 h. After cooling, the obtained product was collected by filtration and crystallized from the mixed solvents of ethanol and di­methyl­formamide (DMF) (3:2 *v*/*v*). The overall reaction scheme is shown in Fig. 3 ▸.

Yield: 78%; m.p. 483–485 K.. Analysis for C_28_H_22_FN_2_OS: MS (*m*/*z*) 468.15 (*M* ^+^ + 1). ^1^H NMR (400 MHz, CDCl_3_): δ 2.27 (*s*, 3H) 2.79 (*s*, 1H, triple-bonded C—H), 3.09 (*dd*, 1H, *J*_AX_ = 18.2, *J*_AB_ = 5.8 Hz), 3.83 (*dd*, 1H, *J*_XA_ = 18.6, *J*_XB_ = 13.2 Hz), 4.40 (*s*, 2H, O—CH_2_), 5.34 (*dd*, 1H, *J*_BA_ = 5.8, *J*_BX_ = 12.8 Hz), 7.08 (*dd*, 2H, *J* = 8.5 Hz, Ar-H), 7.13 (*dd*, 2H, *J* = 8.1 Hz, Ar-H), 7.26 (*dd*, 2H, *J* = 8.8 Hz, Ar-H), 7.39 (*dd*, 2H, *J* = 8.5 Hz, Ar-H), 7.41 (*dd*, 2H, *J* = 8.8 Hz, Ar-H), 7.69 (*dd*, 2H, *J* = 8.1 Hz, Ar-H), 8.09 (*s*, 1*H*-thia­zole-H).

## Refinement

Crystal data, data collection and structure refinement details for the title compound are summarized in Table 2 ▸.

## Supplementary Material

Crystal structure: contains datablock(s) I. DOI: 10.1107/S2414314622010033/bt4127sup1.cif

Structure factors: contains datablock(s) I. DOI: 10.1107/S2414314622010033/bt4127Isup2.hkl

Supporting information file. DOI: 10.1107/S2414314622010033/bt4127Isup3.cml

CCDC reference: 2212832

Additional supporting information:  crystallographic information; 3D view; checkCIF report

## Figures and Tables

**Table 1:** Hydrogen-bond geometry (Å, °)

*D*—H⋯*A*	*D*—H	H⋯*A*	*D*⋯*A*	*D*—H⋯*A*
C12—H12⋯N3	0.95	2.52	2.849 (2)	100
C16—H16⋯S1^i^	0.95	3.10	3.9986 (19)	159
C21—H21⋯N2	0.95	2.55	2.853 (2)	99
C22—H22⋯F1^ii^	0.95	2.53	3.245 (2)	132

Symmetry codes: (i) 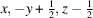 ; (ii) 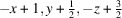 .

**Table 2:** Experimental details

Crystal data	
Chemical formula	C_28_H_22_FN_3_OS
*M*_r_	467.54
Crystal system, space group	Monoclinic, *P*2_1_/*c*
Temperature (K)	100
*a*, *b*, *c* (Å)	10.7859 (13), 14.5638 (16), 14.9956 (14)
β (°)	97.144 (3)
*V* (Å^3^)	2337.3 (4)
*Z*	4
Radiation type	Mo *K*α
μ (mm^−1^)	0.17
Crystal size (mm)	0.24 × 0.17 × 0.12

Data collection	
Diffractometer	Bruker APEXII CCD
Absorption correction	Multi-scan (*SADABS*; Sheldrick, 2003 ▸)
*T*_min_, *T*_max_	0.667, 0.740
No. of measured, independent and observed [*I* > 2σ(*I*)] reflections	34158, 5339, 3999
*R*_int_	0.055
(sin θ/λ)_max_ (Å^−1^)	0.649

Refinement	
*R*[*F* ^2^ > 2σ(*F* ^2^)], *wR*(*F* ^2^), *S*	0.043, 0.124, 1.05
No. of reflections	5339
No. of parameters	312
H-atom treatment	H atoms treated by a mixture of independent and constrained refinement
Δρ_max_, Δρ_min_ (e Å^−3^)	0.21, −0.25

Computer programs: *APEX2* (Bruker, 2005 ▸), *SHELXT* (Sheldrick, 2015*a* ▸), *SHELXL2018* (Sheldrick, 2015*b* ▸) and *OLEX2* (Dolomanov *et al.*, 2009 ▸).

## full crystallographic data

### Crystal data

**Table:**

C_28_H_22_FN_3_OS	*F*(000) = 976
*M**_r_* = 467.54	*D*_x_ = 1.329 Mg m^−^^3^
Monoclinic, *P*2_1_/*c*	Mo *K*α radiation, λ = 0.71073 Å
*a* = 10.7859 (13) Å	Cell parameters from 8134 reflections
*b* = 14.5638 (16) Å	θ = 2.3–19.4°
*c* = 14.9956 (14) Å	µ = 0.17 mm^−^^1^
β = 97.144 (3)°	*T* = 100 K
*V* = 2337.3 (4) Å^3^	Prism, pale yellow
*Z* = 4	0.24 × 0.17 × 0.12 mm

### Data collection

**Table:**

Bruker APEXII CCD diffractometer	3999 reflections with *I* > 2σ(*I*)
ω & φ scans	*R*_int_ = 0.055
Absorption correction: multi-scan (SADABS; Sheldrick, 2003)	θ_max_ = 27.5°, θ_min_ = 2.4°
*T*_min_ = 0.667, *T*_max_ = 0.740	*h* = −13→13
34158 measured reflections	*k* = −18→18
5339 independent reflections	*l* = −19→18

### Refinement

**Table:**

Refinement on *F*^2^	Primary atom site location: structure-invariant direct methods
Least-squares matrix: full	Hydrogen site location: mixed
*R*[*F*^2^ > 2σ(*F*^2^)] = 0.043	H atoms treated by a mixture of independent and constrained refinement
*wR*(*F*^2^) = 0.124	*w* = 1/[σ^2^(*F*_o_^2^) + (0.0555*P*)^2^ + 0.4922*P*] where *P* = (*F*_o_^2^ + 2*F*_c_^2^)/3
*S* = 1.05	(Δ/σ)_max_ = 0.001
5339 reflections	Δρ_max_ = 0.21 e Å^−^^3^
312 parameters	Δρ_min_ = −0.24 e Å^−^^3^
0 restraints	

### Special details

**Table:**

Geometry. All esds (except the esd in the dihedral angle between two l.s. planes) are estimated using the full covariance matrix. The cell esds are taken into account individually in the estimation of esds in distances, angles and torsion angles; correlations between esds in cell parameters are only used when they are defined by crystal symmetry. An approximate (isotropic) treatment of cell esds is used for estimating esds involving l.s. planes.

### Fractional atomic coordinates and isotropic or equivalent isotropic displacement parameters (Å^2^)

**Table:**

	*x*	*y*	*z*	*U*_iso_*/*U*_eq_	
S1	0.41974 (4)	0.50137 (3)	0.68772 (3)	0.05135 (14)	
F1	0.30040 (14)	0.00083 (9)	0.45510 (11)	0.0947 (5)	
O1	1.05115 (12)	0.28517 (9)	1.05004 (8)	0.0620 (4)	
N1	0.55591 (13)	0.35607 (10)	0.64338 (9)	0.0504 (3)	
N2	0.62314 (12)	0.36498 (9)	0.72765 (9)	0.0458 (3)	
N3	0.40086 (11)	0.41449 (9)	0.53590 (9)	0.0416 (3)	
C1	0.71955 (14)	0.31161 (11)	0.73107 (11)	0.0429 (4)	
C2	0.72870 (16)	0.25989 (13)	0.64542 (11)	0.0519 (4)	
H2A	0.725916	0.192751	0.655328	0.062*	
H2B	0.806651	0.275473	0.620224	0.062*	
C3	0.61252 (15)	0.29284 (11)	0.58290 (11)	0.0453 (4)	
H3	0.639256	0.327907	0.531225	0.054*	
C4	0.46340 (14)	0.41667 (10)	0.61583 (11)	0.0421 (3)	
C5	0.30581 (16)	0.53433 (12)	0.60280 (11)	0.0501 (4)	
H5	0.248195	0.582902	0.607499	0.060*	
C6	0.30918 (15)	0.48190 (10)	0.52848 (11)	0.0415 (3)	
C7	0.22498 (15)	0.48815 (10)	0.44307 (11)	0.0428 (4)	
C8	0.11627 (19)	0.53946 (15)	0.43594 (13)	0.0641 (5)	
H8	0.096611	0.573810	0.486179	0.077*	
C9	0.0357 (2)	0.54138 (16)	0.35642 (14)	0.0711 (6)	
H9	−0.038797	0.576647	0.353696	0.085*	
C10	0.06012 (18)	0.49389 (12)	0.28146 (12)	0.0542 (4)	
C11	0.16914 (18)	0.44361 (13)	0.28824 (12)	0.0554 (4)	
H11	0.189312	0.410573	0.237352	0.066*	
C12	0.25025 (16)	0.44011 (12)	0.36778 (11)	0.0499 (4)	
H12	0.324148	0.404195	0.370509	0.060*	
C13	−0.0308 (2)	0.49575 (16)	0.19603 (14)	0.0742 (6)	
H13A	−0.115982	0.487080	0.210809	0.111*	
H13B	−0.010253	0.446321	0.156014	0.111*	
H13C	−0.024930	0.555058	0.165965	0.111*	
C14	0.52458 (14)	0.21655 (11)	0.54827 (10)	0.0417 (3)	
C15	0.50313 (16)	0.19735 (12)	0.45752 (11)	0.0513 (4)	
H15	0.540276	0.234834	0.416283	0.062*	
C16	0.42798 (18)	0.12400 (13)	0.42561 (13)	0.0605 (5)	
H16	0.414806	0.109938	0.363286	0.073*	
C17	0.37364 (18)	0.07275 (13)	0.48627 (15)	0.0615 (5)	
C18	0.3914 (2)	0.08969 (13)	0.57641 (15)	0.0645 (5)	
H18	0.352303	0.052768	0.617046	0.077*	
C19	0.46802 (17)	0.16219 (12)	0.60694 (12)	0.0537 (4)	
H19	0.481996	0.174834	0.669533	0.064*	
C20	0.80833 (14)	0.30375 (11)	0.81275 (10)	0.0425 (3)	
C21	0.79227 (15)	0.35436 (12)	0.88945 (11)	0.0484 (4)	
H21	0.722761	0.394575	0.888410	0.058*	
C22	0.87487 (16)	0.34712 (13)	0.96621 (11)	0.0515 (4)	
H22	0.862460	0.382276	1.017719	0.062*	
C23	0.97723 (15)	0.28819 (12)	0.96889 (11)	0.0459 (4)	
C24	0.99600 (16)	0.23779 (12)	0.89378 (11)	0.0503 (4)	
H24	1.065768	0.197771	0.895145	0.060*	
C25	0.91183 (15)	0.24615 (12)	0.81614 (11)	0.0493 (4)	
H25	0.925194	0.211853	0.764298	0.059*	
C26	1.15665 (16)	0.22557 (13)	1.05765 (11)	0.0520 (4)	
H26A	1.221624	0.250122	1.022969	0.062*	
H26B	1.131967	0.163786	1.034290	0.062*	
C27	1.20376 (17)	0.22073 (13)	1.15271 (12)	0.0553 (4)	
C28	1.2393 (2)	0.21588 (18)	1.22951 (16)	0.0804 (7)	
H28	1.256 (3)	0.209 (2)	1.2914 (19)	0.121 (10)*	

### Atomic displacement parameters (Å^2^)

**Table:**

	*U*^11^	*U*^22^	*U*^33^	*U*^12^	*U*^13^	*U*^23^
S1	0.0563 (3)	0.0476 (2)	0.0466 (2)	0.00514 (19)	−0.00782 (18)	−0.00366 (18)
F1	0.0895 (9)	0.0677 (8)	0.1199 (12)	−0.0298 (7)	−0.0144 (8)	−0.0172 (7)
O1	0.0650 (8)	0.0700 (8)	0.0460 (7)	0.0306 (7)	−0.0132 (6)	−0.0083 (6)
N1	0.0508 (8)	0.0449 (8)	0.0497 (8)	0.0081 (6)	−0.0162 (6)	−0.0063 (6)
N2	0.0465 (7)	0.0418 (7)	0.0454 (7)	0.0022 (6)	−0.0085 (6)	−0.0006 (6)
N3	0.0399 (7)	0.0379 (7)	0.0445 (7)	−0.0016 (5)	−0.0049 (5)	0.0027 (5)
C1	0.0404 (8)	0.0409 (8)	0.0454 (9)	−0.0008 (6)	−0.0026 (6)	0.0003 (7)
C2	0.0448 (9)	0.0569 (10)	0.0511 (10)	0.0043 (8)	−0.0060 (7)	−0.0070 (8)
C3	0.0450 (8)	0.0438 (9)	0.0447 (9)	0.0004 (7)	−0.0039 (7)	−0.0011 (7)
C4	0.0401 (8)	0.0359 (8)	0.0477 (9)	−0.0024 (6)	−0.0045 (6)	0.0026 (6)
C5	0.0523 (9)	0.0466 (9)	0.0487 (10)	0.0080 (8)	−0.0042 (7)	0.0019 (7)
C6	0.0406 (8)	0.0371 (8)	0.0454 (8)	−0.0012 (6)	−0.0003 (6)	0.0063 (6)
C7	0.0427 (8)	0.0383 (8)	0.0458 (9)	−0.0022 (6)	−0.0012 (7)	0.0060 (6)
C8	0.0623 (11)	0.0692 (12)	0.0569 (11)	0.0230 (10)	−0.0086 (9)	−0.0102 (9)
C9	0.0638 (12)	0.0771 (14)	0.0658 (13)	0.0250 (11)	−0.0178 (10)	−0.0050 (11)
C10	0.0587 (10)	0.0510 (10)	0.0489 (10)	−0.0079 (8)	−0.0096 (8)	0.0093 (8)
C11	0.0636 (11)	0.0578 (11)	0.0435 (9)	−0.0051 (9)	0.0017 (8)	0.0012 (8)
C12	0.0483 (9)	0.0517 (10)	0.0488 (9)	0.0014 (7)	0.0032 (7)	0.0040 (7)
C13	0.0806 (15)	0.0761 (14)	0.0581 (12)	−0.0112 (11)	−0.0225 (10)	0.0095 (10)
C14	0.0432 (8)	0.0388 (8)	0.0408 (8)	0.0043 (6)	−0.0039 (6)	0.0010 (6)
C15	0.0566 (10)	0.0523 (10)	0.0425 (9)	−0.0013 (8)	−0.0036 (7)	0.0028 (7)
C16	0.0681 (12)	0.0584 (11)	0.0489 (10)	0.0040 (9)	−0.0161 (9)	−0.0086 (8)
C17	0.0570 (11)	0.0439 (10)	0.0800 (14)	−0.0070 (8)	−0.0058 (10)	−0.0063 (9)
C18	0.0711 (12)	0.0482 (10)	0.0755 (13)	−0.0109 (9)	0.0145 (10)	0.0017 (9)
C19	0.0651 (11)	0.0480 (9)	0.0475 (10)	−0.0006 (8)	0.0052 (8)	0.0003 (7)
C20	0.0396 (8)	0.0431 (8)	0.0429 (8)	0.0008 (6)	−0.0021 (6)	0.0016 (6)
C21	0.0442 (8)	0.0516 (9)	0.0474 (9)	0.0145 (7)	−0.0021 (7)	−0.0003 (7)
C22	0.0535 (9)	0.0570 (10)	0.0427 (9)	0.0163 (8)	0.0001 (7)	−0.0055 (7)
C23	0.0458 (8)	0.0488 (9)	0.0407 (8)	0.0091 (7)	−0.0041 (6)	0.0016 (7)
C24	0.0450 (9)	0.0550 (10)	0.0488 (9)	0.0161 (8)	−0.0026 (7)	−0.0037 (8)
C25	0.0482 (9)	0.0518 (10)	0.0458 (9)	0.0094 (7)	−0.0020 (7)	−0.0072 (7)
C26	0.0464 (9)	0.0547 (10)	0.0517 (10)	0.0109 (8)	−0.0064 (7)	0.0000 (8)
C27	0.0515 (10)	0.0570 (10)	0.0539 (11)	0.0114 (8)	−0.0068 (8)	0.0014 (8)
C28	0.0879 (16)	0.0896 (17)	0.0579 (14)	0.0258 (13)	−0.0137 (11)	0.0045 (12)

### Geometric parameters (Å, º)

**Table:**

S1—C4	1.7412 (17)	C11—C12	1.390 (2)
S1—C5	1.7241 (17)	C12—H12	0.9500
F1—C17	1.359 (2)	C13—H13A	0.9800
O1—C23	1.3701 (19)	C13—H13B	0.9800
O1—C26	1.4243 (19)	C13—H13C	0.9800
N1—N2	1.3827 (17)	C14—C15	1.380 (2)
N1—C3	1.477 (2)	C14—C19	1.381 (2)
N1—C4	1.357 (2)	C15—H15	0.9500
N2—C1	1.294 (2)	C15—C16	1.390 (2)
N3—C4	1.3005 (19)	C16—H16	0.9500
N3—C6	1.388 (2)	C16—C17	1.364 (3)
C1—C2	1.503 (2)	C17—C18	1.364 (3)
C1—C20	1.462 (2)	C18—H18	0.9500
C2—H2A	0.9900	C18—C19	1.383 (3)
C2—H2B	0.9900	C19—H19	0.9500
C2—C3	1.545 (2)	C20—C21	1.395 (2)
C3—H3	1.0000	C20—C25	1.392 (2)
C3—C14	1.510 (2)	C21—H21	0.9500
C5—H5	0.9500	C21—C22	1.369 (2)
C5—C6	1.355 (2)	C22—H22	0.9500
C6—C7	1.478 (2)	C22—C23	1.395 (2)
C7—C8	1.383 (2)	C23—C24	1.380 (2)
C7—C12	1.384 (2)	C24—H24	0.9500
C8—H8	0.9500	C24—C25	1.390 (2)
C8—C9	1.386 (3)	C25—H25	0.9500
C9—H9	0.9500	C26—H26A	0.9900
C9—C10	1.373 (3)	C26—H26B	0.9900
C10—C11	1.378 (3)	C26—C27	1.454 (2)
C10—C13	1.513 (2)	C27—C28	1.170 (3)
C11—H11	0.9500	C28—H28	0.93 (3)
			
C5—S1—C4	87.87 (8)	C10—C13—H13B	109.5
C23—O1—C26	117.49 (13)	C10—C13—H13C	109.5
N2—N1—C3	114.19 (12)	H13A—C13—H13B	109.5
C4—N1—N2	119.95 (13)	H13A—C13—H13C	109.5
C4—N1—C3	124.26 (13)	H13B—C13—H13C	109.5
C1—N2—N1	107.90 (13)	C15—C14—C3	120.60 (15)
C4—N3—C6	109.84 (13)	C15—C14—C19	118.61 (15)
N2—C1—C2	113.90 (13)	C19—C14—C3	120.74 (14)
N2—C1—C20	121.29 (14)	C14—C15—H15	119.5
C20—C1—C2	124.81 (14)	C14—C15—C16	120.95 (17)
C1—C2—H2A	111.1	C16—C15—H15	119.5
C1—C2—H2B	111.1	C15—C16—H16	120.9
C1—C2—C3	103.20 (13)	C17—C16—C15	118.14 (17)
H2A—C2—H2B	109.1	C17—C16—H16	120.9
C3—C2—H2A	111.1	F1—C17—C16	118.16 (19)
C3—C2—H2B	111.1	F1—C17—C18	118.96 (19)
N1—C3—C2	100.79 (12)	C18—C17—C16	122.87 (17)
N1—C3—H3	109.8	C17—C18—H18	121.0
N1—C3—C14	112.32 (13)	C17—C18—C19	118.08 (18)
C2—C3—H3	109.8	C19—C18—H18	121.0
C14—C3—C2	114.04 (14)	C14—C19—C18	121.33 (17)
C14—C3—H3	109.8	C14—C19—H19	119.3
N1—C4—S1	121.16 (12)	C18—C19—H19	119.3
N3—C4—S1	116.01 (12)	C21—C20—C1	120.91 (14)
N3—C4—N1	122.82 (15)	C25—C20—C1	121.08 (14)
S1—C5—H5	124.4	C25—C20—C21	118.01 (14)
C6—C5—S1	111.16 (13)	C20—C21—H21	119.4
C6—C5—H5	124.4	C22—C21—C20	121.19 (15)
N3—C6—C7	117.90 (14)	C22—C21—H21	119.4
C5—C6—N3	115.10 (14)	C21—C22—H22	119.9
C5—C6—C7	126.98 (15)	C21—C22—C23	120.10 (15)
C8—C7—C6	121.59 (16)	C23—C22—H22	119.9
C8—C7—C12	117.46 (16)	O1—C23—C22	114.61 (14)
C12—C7—C6	120.92 (15)	O1—C23—C24	125.41 (14)
C7—C8—H8	119.6	C24—C23—C22	119.97 (15)
C7—C8—C9	120.82 (18)	C23—C24—H24	120.3
C9—C8—H8	119.6	C23—C24—C25	119.35 (15)
C8—C9—H9	119.0	C25—C24—H24	120.3
C10—C9—C8	122.04 (18)	C20—C25—H25	119.3
C10—C9—H9	119.0	C24—C25—C20	121.37 (15)
C9—C10—C11	117.13 (16)	C24—C25—H25	119.3
C9—C10—C13	120.92 (19)	O1—C26—H26A	110.4
C11—C10—C13	121.93 (19)	O1—C26—H26B	110.4
C10—C11—H11	119.2	O1—C26—C27	106.64 (14)
C10—C11—C12	121.54 (17)	H26A—C26—H26B	108.6
C12—C11—H11	119.2	C27—C26—H26A	110.4
C7—C12—C11	120.99 (16)	C27—C26—H26B	110.4
C7—C12—H12	119.5	C28—C27—C26	178.5 (2)
C11—C12—H12	119.5	C27—C28—H28	171.9 (19)
C10—C13—H13A	109.5		
			
S1—C5—C6—N3	0.04 (19)	C4—N3—C6—C7	−177.89 (13)
S1—C5—C6—C7	178.66 (13)	C5—S1—C4—N1	−177.59 (15)
F1—C17—C18—C19	178.88 (18)	C5—S1—C4—N3	1.24 (13)
O1—C23—C24—C25	178.61 (17)	C5—C6—C7—C8	−12.2 (3)
N1—N2—C1—C2	0.80 (19)	C5—C6—C7—C12	170.00 (17)
N1—N2—C1—C20	−178.53 (14)	C6—N3—C4—S1	−1.41 (17)
N1—C3—C14—C15	129.54 (16)	C6—N3—C4—N1	177.40 (15)
N1—C3—C14—C19	−53.1 (2)	C6—C7—C8—C9	−177.19 (18)
N2—N1—C3—C2	1.52 (18)	C6—C7—C12—C11	177.95 (15)
N2—N1—C3—C14	123.29 (14)	C7—C8—C9—C10	−0.7 (4)
N2—N1—C4—S1	−4.6 (2)	C8—C7—C12—C11	0.0 (3)
N2—N1—C4—N3	176.69 (14)	C8—C9—C10—C11	0.0 (3)
N2—C1—C2—C3	0.14 (19)	C8—C9—C10—C13	178.8 (2)
N2—C1—C20—C21	−0.6 (2)	C9—C10—C11—C12	0.8 (3)
N2—C1—C20—C25	179.91 (16)	C10—C11—C12—C7	−0.8 (3)
N3—C6—C7—C8	166.41 (17)	C12—C7—C8—C9	0.7 (3)
N3—C6—C7—C12	−11.4 (2)	C13—C10—C11—C12	−178.06 (17)
C1—C2—C3—N1	−0.93 (16)	C14—C15—C16—C17	1.5 (3)
C1—C2—C3—C14	−121.48 (15)	C15—C14—C19—C18	0.1 (3)
C1—C20—C21—C22	179.82 (16)	C15—C16—C17—F1	−179.82 (17)
C1—C20—C25—C24	−179.47 (16)	C15—C16—C17—C18	−1.0 (3)
C2—C1—C20—C21	−179.83 (16)	C16—C17—C18—C19	0.0 (3)
C2—C1—C20—C25	0.7 (3)	C17—C18—C19—C14	0.4 (3)
C2—C3—C14—C15	−116.59 (17)	C19—C14—C15—C16	−1.1 (3)
C2—C3—C14—C19	60.7 (2)	C20—C1—C2—C3	179.44 (15)
C3—N1—N2—C1	−1.52 (19)	C20—C21—C22—C23	−0.2 (3)
C3—N1—C4—S1	−169.30 (12)	C21—C20—C25—C24	1.0 (3)
C3—N1—C4—N3	11.9 (3)	C21—C22—C23—O1	−178.37 (16)
C3—C14—C15—C16	176.31 (16)	C21—C22—C23—C24	0.7 (3)
C3—C14—C19—C18	−177.27 (17)	C22—C23—C24—C25	−0.3 (3)
C4—S1—C5—C6	−0.66 (14)	C23—O1—C26—C27	−168.80 (16)
C4—N1—N2—C1	−167.73 (15)	C23—C24—C25—C20	−0.5 (3)
C4—N1—C3—C2	167.05 (15)	C25—C20—C21—C22	−0.7 (3)
C4—N1—C3—C14	−71.2 (2)	C26—O1—C23—C22	179.37 (16)
C4—N3—C6—C5	0.9 (2)	C26—O1—C23—C24	0.4 (3)

### Hydrogen-bond geometry (Å, º)

**Table:**

*D*—H···*A*	*D*—H	H···*A*	*D*···*A*	*D*—H···*A*
C12—H12···N3	0.95	2.52	2.849 (2)	100
C16—H16···S1^i^	0.95	3.10	3.9986 (19)	159
C21—H21···N2	0.95	2.55	2.853 (2)	99
C22—H22···F1^ii^	0.95	2.53	3.245 (2)	132

Symmetry codes: (i) *x*, −*y*+1/2, *z*−1/2; (ii) −*x*+1, *y*+1/2, −*z*+3/2.
